# Biosourced Poly(lactic acid)/polyamide-11 Blends: Effect of an Elastomer on the Morphology and Mechanical Properties

**DOI:** 10.3390/molecules27206819

**Published:** 2022-10-12

**Authors:** Ali Fazli, Denis Rodrigue

**Affiliations:** Department of Chemical Engineering, Laval University, Quebec, QC G1V 0A6, Canada

**Keywords:** biobased blends, poly(lactic acid), polyamide-11, morphology, interfacial tension, impact strength

## Abstract

Fully biobased polylactide (PLA)/polyamide-11 (PA11) blends were prepared by melt mixing with an elastomer intermediate phase to address the low elasticity and brittleness of PLA blends. The incorporation of a biobased elastomer made of poly(butylene adipate-co-terephthalate) (PBAT) and polyethylene oxide (PEO) copolymers was found to change the rigid interface between PLA and PA11 into a much more elastic/deformable one as well as promote interfacial compatibility. The interfacial tension of the polymer pairs and spreading coefficients revealed a high tendency of PEO to spread at the PLA/PA11 interface, resulting in a complete wetting regime (interfacial tension of 0.56 mN/m). A fully percolated rubbery phase (PEO) layer at the PLA/PA11 interface with enhanced interfacial interactions and PLA chain mobility contributed to a better distribution of the stress around the dispersed phase, leading to shear yielding of the matrix. The results also show that both the morphological modification and improved compatibility upon PEO addition (up to 20 wt %) contributed to the improved elongation at break (up to 104%) and impact strength (up to 292%) of the ternary PLA/PA11/PEO blends to obtain a super-tough multiphase system.

## 1. Introduction

Poly(lactic acid) (PLA), as an aliphatic biobased polyester made from fermented plant starch, has attracted significant attention in the industry and academia as an alternative material to petroleum-based plastics to develop sustainable polymeric materials [1,2]. PLA is one of the most produced and consumed biobased, biodegradable and biocompatible plastics because of its high tensile strength (59 MPa) and tensile modulus (1280 MPa) combined with good biocompatibility, transparency, compostability and processability [3,4]. Despite these performances and the wide availability of different grades, PLA has low heat distortion temperature (HDT) (55 °C), inherent brittleness (less than 10% elongation at break) and poor impact strength (Notch Izod impact strength of 26 J/m), limiting its practical applications in biomedical, packaging and automotive industries [3,5]. To overcome these limitations, several studies reported on different options to improve the heat resistance and impact strength of PLA. The most common methods are based on copolymerization [6], plasticization [7] and physical blending [8]. For the latter, PLA has been melt blended with other bioplastics, such as polyhydroxyalkanoate (PHA) [9], poly(3-hydroxybutyrate-co-3-hydroxyvalerate) (PHBV) [10], poly(butylene adipate-co-terephthalate) (PBAT) [11] and poly(butylene succinate) (PBS) [12], which are seen as practical and economical approaches to overcome the PLA shortcomings.

On the other hand, polyamide-11 (PA11), as a bioplastic produced from castor oil, has good HDT, impact strength and elongation-at-break, chemical resistance, and excellent dimensional stability, exhibiting a similar glass transition temperature (*T_g_* ≈ 45 °C) and melting point (*T_m_* ≈ 190 °C) to PLA [13,14]. However, the superior mechanical performance of PA11 is attributed to intermolecular hydrogen bonding in the crystalline and amorphous state [7].

Few studies have been carried out on PLA/PA11 blends as fully biobased materials for applications such as medical implants and devices, food packaging, agricultural films, etc. Feng and Ye [15] observed a partial miscibility between PLA and PA11 due to hydrogen bonding between the amino (NH) groups of PA11 and the carbonyl (CO) groups of PLA. Despite interfacial interactions between PLA and PA11, Heshmati and Favis [7] reported very low elongation at break (6%) and impact strength (11 J/m) of a binary PLA/PA11 (50/50) blend in line with similar studies highlighting the necessity to perform compatibilization for PLA-based blends [16,17].

In general, compatibilization studies focused on the development of binary polymer systems (PLA = matrix), while the presence of an intermediate phase in ternary blends is more effective to provide good interfacial interactions between the phases to generate super-tough PLA-based systems [18]. For example, Mehrabi et al. [19] developed highly tough PLA-based ternary systems by the inclusion of a core–shell impact modifier based on polybutadiene-*g*-poly(styrene-co-acrylonitrile) (PB-*g*-SAN) and poly(methyl methacrylate) (PMMA). The improved dispersion state of PB-*g*-SAN terpolymer and its interfacial interactions with PLA as a result of the partial miscibility of PMMA with PLA and terpolymer (PMMA miscibility with SAN) was shown to increase the impact strength and elongation at break of PLA/PB-*g*-SAN/PMMA (45/30/25) from 25.6 J/m and 6% to 500 J/m and 80%, respectively, upon the addition of 25% PMMA. Following this concept, Zhang et al. [20] optimized the blend composition of multiphase systems based on PLA/ethylenemethyl acrylate-glycidyl methacrylate (EMA-GMA)/poly(ether-b-amide) (PEBA) (70/20/10) where PEBA encapsulated the EMA-GMA, exhibiting a core–shell structure leading to an impact strength of 410 J/m with an elongation at break of 72.7%. However, the majority of studies on ternary immiscible polymer blends deal with core–shell morphologies, while multiphase systems with co-continuous and tri-continuous phase morphology also have the potential to modify the mechanical properties, i.e., the impact and tensile properties of PLA blends [21]. Controlling the morphologies and interfacial properties (complex interaction between all the components) can lead to appropriate stress transfer between the components by the creation of intermediate phases to generate superior properties in multicomponent systems [19,22]. Li and Shimizu [23] reported on PLA impact modification by blending with acrylonitrile–butadiene–styrene copolymer (ABS) with the help of 5% styrene/acrylonitrile/glycidyl methacrylate copolymer (SAN-GMA) as a reactive compatibilizer. The epoxide groups of SAN-GMA can react either with –COOH or –OH on the PLA end groups to form an intermediate layer, decreasing the ABS domain size with a narrow size distribution improving the mixing state (uniformity). Thus, PLA/ABS (50/50) compatibilized with 5 wt % SAN-GMA could undergo stretching up to 23.5% before breaking and showed higher impact strength (162.8 kJ/m^2^) compared with the neat PLA/ABS (70/30) blend (48.3 kJ/m^2^). Generally, based on the equilibrium of interfacial forces in multicomponent immiscible systems and the values of the spreading coefficient, complete wetting or partial wetting may occur, leading to the generation of core–shell, tri-continuous structures (complete wetting) or multiple stacked morphologies where none of the phases completely spread at the interface of the other two (partial wetting) [24]. According to Zolali and Favis [22], the development of a tri-continuous morphology contributed to a noticeable improvement in the impact strength of ternary PLA-based blends. Possible interactions between the polyether blocks of PEBA with PLA and its amide affinity toward PA11 decreased the interfacial tension of ternary PLA/PA11/PEBA blends, and a positive spreading coefficient (*λ_PLA/PEBA/PA_* = +0.3) suggested the complete assembling of the PEBA elastomer at the PLA/PA11 interface. The substitution of a rigid PLA/PA11 interface with a thick (350 nm) deformable interface of PEBA led to the development of a tri-continuous structure of interconnected phases, increasing the impact strength by about 8 times (from 17.3 to 142.4 J/m) in the ternary PLA/PA11/PEBA (45/45/10) system [22]. In another work, Ravati et al. [25] observed a tri-continuous morphology for a PLA/PBAT/PBS (33/33/33) blend with a positive spreading coefficient (*λ*_PLA/PBAT/PBS_ = 0.3  ±  0.2 mN/m), indicating that the PBAT phase completely wet the interface between PLA and PBS by locating between both phases, leading to high impact strength (271 J/m) and elongation at break (567.9%) due to enhanced shear yielding and plastic deformation of the PLA matrix.

Despite a number of studies addressing the issues of low deformation and the poor impact resistance of PLA blends without sacrificing stiffness and strength, it is still not clear how the phase structure and phase interaction in multicomponent blends contribute to the impact modification of ternary blends. The objective of this article is to report on the effect of adding an intermediate elastomer phase and the blend composition on the morphology development of fully biobased PLA/PA11 blends prepared by melt blending. In particular, polyethylene oxide (PEO) and PBAT are used to modify the blends. For this purpose, a theoretical prediction of interfacial adhesion between the components of ternary systems and spreading coefficients are evaluated to assess their potential to locate at the interface and generate a partial or complete wetting morphology, forming a core–shell or tri-continuous structure. Based on the blend structures produced, their effect on the tensile and impact properties is reported.

## 2. Results and Discussion

### 2.1. Theoretical Prediction of Interfacial Tension

**Figure 1:**
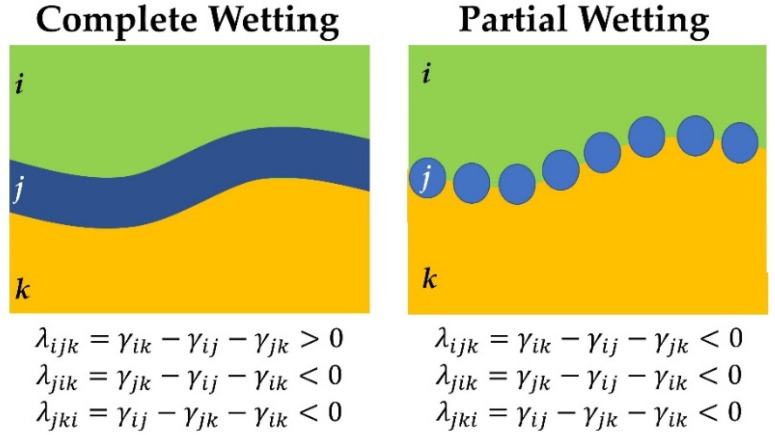
Schematic representation of the blend structure associated with complete and partial wetting morphologies in ternary polymer blends.

The morphology of multiphase systems thermodynamically depends on the interfacial tension between polymer pairs [26]. Torza and Mason [27] were the first to use the concept of a spreading coefficient to predict the phase morphology and wetting phenomenon of ternary blends through the calculation of spreading coefficients. The spreading coefficient model is a commonly used theoretical model to evaluate the tendency of a component to segregate the other two components and to predict the morphology of immiscible ternary blends [27]. This method is based on the interfacial tensions (*γ_ij_*) between each pair of components, which can be calculated via contact angle measurement because of the simplicity and facility of measuring the parameters. The spreading theory first was developed by Harkins in the 1920s to predict the spreading coefficients and wetting regime of ternary polymer systems by the following equation [27,28]:

(1) $$\lambda_{ijk}=\gamma_{ik}-\gamma_{ij}-\gamma_{jk}$$

where *λ* is the spreading coefficient and *γ* represents the interfacial tensions between the polymer pairs indicated by sub-indices. Here, the spreading coefficient (*λ_ijk_*) predicts the tendency of component (*j*) to segregate the other phases and be located at the interface between components (*i*) and (*k*) [28]. For a ternary blend, three spreading coefficients are required to predict the morphology. A positive value of *λ_ijk_* implies a tendency of one phase (*j*) to separate two other phases (*i*) and (*k*) and form a core–shell or tri-continuous structure (*k* encapsulated by *j*) corresponding to a complete wetting morphology (two-phase contact only). On the other hand, negative values of spreading coefficients imply a three-phase contact, as none of the phases are located fully between the other two, and only one-phase droplets are located at the interface of the other two polymers, implying partial wetting [25,29]. A schematic representation of both wetting scenarios is presented in Figure 1 to better understand the difference between complete wetting and partial wetting regimes in a ternary blend with the corresponding coefficients [24].

The well-known harmonic mean equation can be used to determine the interfacial tension (*γ)* between different components (*i*, *j* and *k*) as follows [26]:

(2) $$\gamma_{ij}=\gamma_{i}+\gamma_{j}-\frac{4\gamma_{i}^{d}\gamma_{j}^{d}}{\gamma_{i}^{d}+\gamma_{j}^{d}}-\frac{4\gamma_{i}^{p}\gamma_{j}^{p}}{\gamma_{i}^{p}+\gamma_{j}^{p}}$$

where *γ*_i_ and *γ*_j_ represent the surface tensions of component (*i*) and (*j*), respectively, while *γ^p^* and *γ^d^* are, respectively, the polar and dispersive components of surface tension ($\gamma_{l}=\gamma_{l}^{d}+\gamma_{l}^{p}$) calculated from the contact angle (Equations (1) and (2)) [26]. Based on data obtained from the sessile drop method, the surface tension of PLA, PA11, PEO and PBAT, as well as the interfacial tension values and the spreading coefficients, are reported in Table 1.

The interfacial tension results for the ternary PLA/PA11/PEO blend show an almost equal affinity of PEO toward PLA to PA11 because of very low and similar interfacial tensions between PEO and the other two polymers as of *γ*_PLA/PEO_ = 1.17 ± 0.5 mN/m and *γ*_PA11/PEO_ = 2.10 ± 0.6 mN/m with respect to *γ*_PLA/PA11_ = 3.65 ± 0.7 mN/m, while PBAT showed slightly higher interfacial values with PLA and PA11 as *γ*_PLA/PBAT_ = 2.87 ± 0.6 mN/m and *γ*_PA11/PBAT_ = 2.38 ± 0.7 mN/m. The incorporation of PEO (plasticizer) is expected to decrease the interfacial tension between PLA and PA11 in line with literature results for which the interfacial tension of PLA/PA11, determined by the in situ Neuman triangle method, decreased from 3.2 to 2.1 ± 0.3 mN/m upon PEO addition (10%) in PLA/PEO/PA11 45/10/45 [13]. The miscibility of PEO with PLA and its increased chain mobility due to the presence of PEO can further enhance the generation of PEO droplets at the interface between PLA and PA11, which underlines the higher thermodynamic driving force of PEO for interfacial wetting than that of PBAT in these ternary systems. In other words, the positive spreading coefficient of *λ_PA11/PEO/PLA_* (0.56 mN/m) indicates the possibility of a high concentration of intermediate phase assembling at the interface and small sizes of the dispersed phases preventing droplet coalescence, which correlates with a higher tendency of PEO to be located between PLA and PA11, thus stabilizing the morphology. This is in agreement with the complete wetting morphology of PEO predicted by the positive spreading coefficients of PLA/PEO/PA11 leading to a continuous layer of elastomer between PLA and PA11 compared to scattered PBAT droplets as a consequence of all three spreading coefficients in PLA/PBAT/PA11 being negative (see Table 1). From Table 1, a negative spreading coefficient (*λ_PLA/PBAT/PA11_ =* −1.6 mN/m) predicts a partial wetting morphology for PLA/PBAT/PA11 ternary blend in agreement with Fu et al. [24] for the prediction of a partial wetting morphology (all spreading coefficients being negative), as the PBAT phase has similar affinity with both PLA and PA11. In agreement with our observation, Zolali and Favis [30] also reported negative spreading coefficients for PLA/PBAT/PA11 blends; thus, all the phases met each other at a three-phase line of contact (partial wetting regime) as a result of the high interfacial tension between each polymer pairs.

### 2.2. Morphological Observation

**Figure 2:**
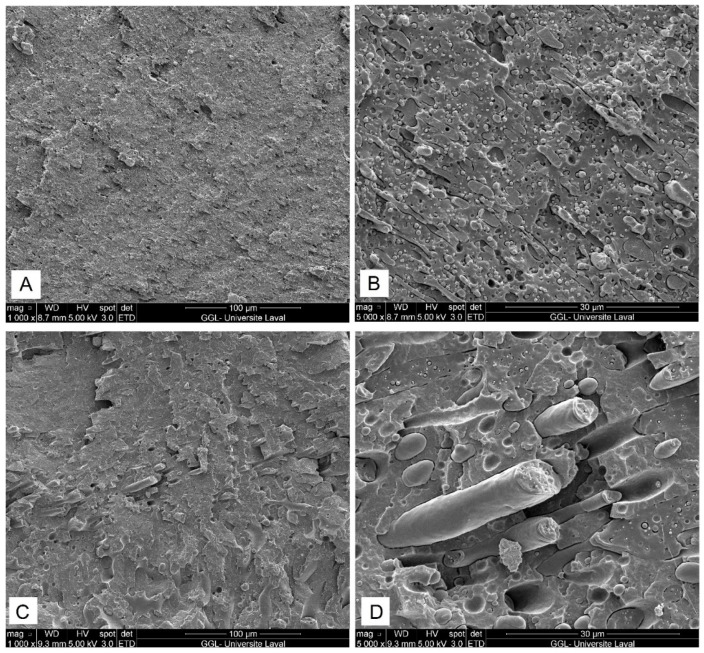
SEM micrographs of PL70/PA30 (**A**,**B**) and PL50/PA50 (**C**,**D**) blends (see Section 3.2 for definition).

The morphological behavior of multiphase systems is of great importance since the overall macroscopic properties highly depend on the morphology development and interface quality. SEM micrographs (Figure 2) present the morphological structure of the binary PL70/PA30 and PL50/PA50 blends depending on the PA11 content. As shown in Figure 2A,B, PL70/PA30 shows a two-phase morphology with a nodular structure (sea-island) of PA11 as the minor phase with low affinity (gaps at the interface) dispersed in the PLA matrix. A clear interfacial region between PLA and PA11 droplets indicates poor interfacial bonding and incompatibility for this system, resulting in dispersion problems and a heterogenous structure in agreement with similar reports [7,31]. Increasing the PA11 content from 30 to 50% contributed to an increase in the size of the dispersed PA11 phase changing from a spherical-like domain in PL70/PA30 (Figure 2A,B) to a more elongated structure in PL50/PA50 (Figure 2C,D). Comparing the phase morphology for different blend compositions, large voids around the spherical PA11 nodules (Figure 2B) and the pull-out of weekly embedded PA11 particles from the PLA matrix during fracture leaving empty cavities (voids) indicate the immiscibility of the binary blend and high interfacial tension between the polymers (high surface energy of the polymer pairs; *γ*_PLA/PA11_ = 3.65 ± 0.7 mN/m), which is a confirmation of their poor compatibility and is in agreement with similar observations [7,31,32].

**Figure 3:**
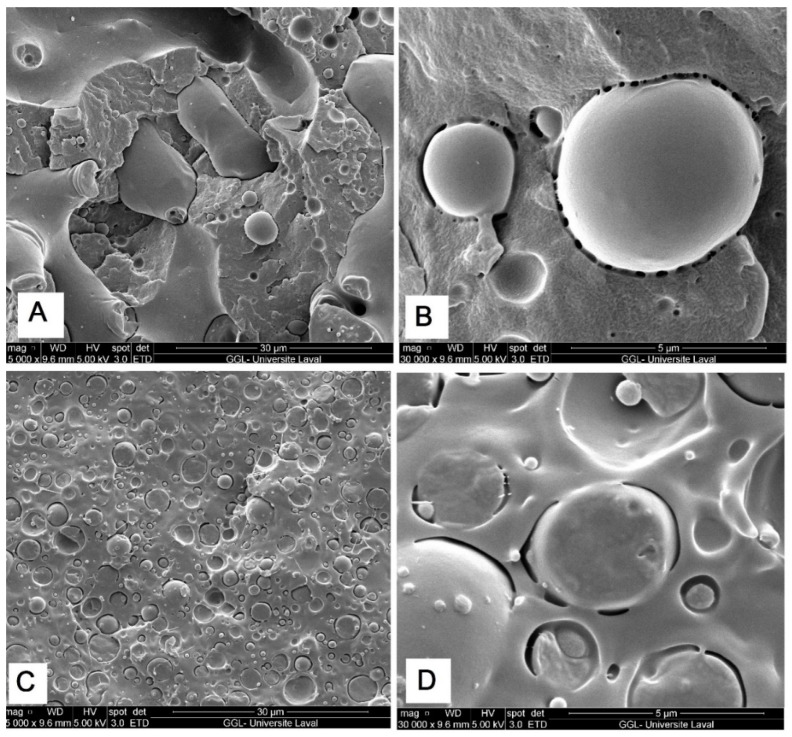
SEM micrographs of PA20/(PBAT20) (**A**,**B**) and PA20/(PEO20) (**C**,**D**) ternary blends (see Section 3.2 for definition).

**Figure 4:**
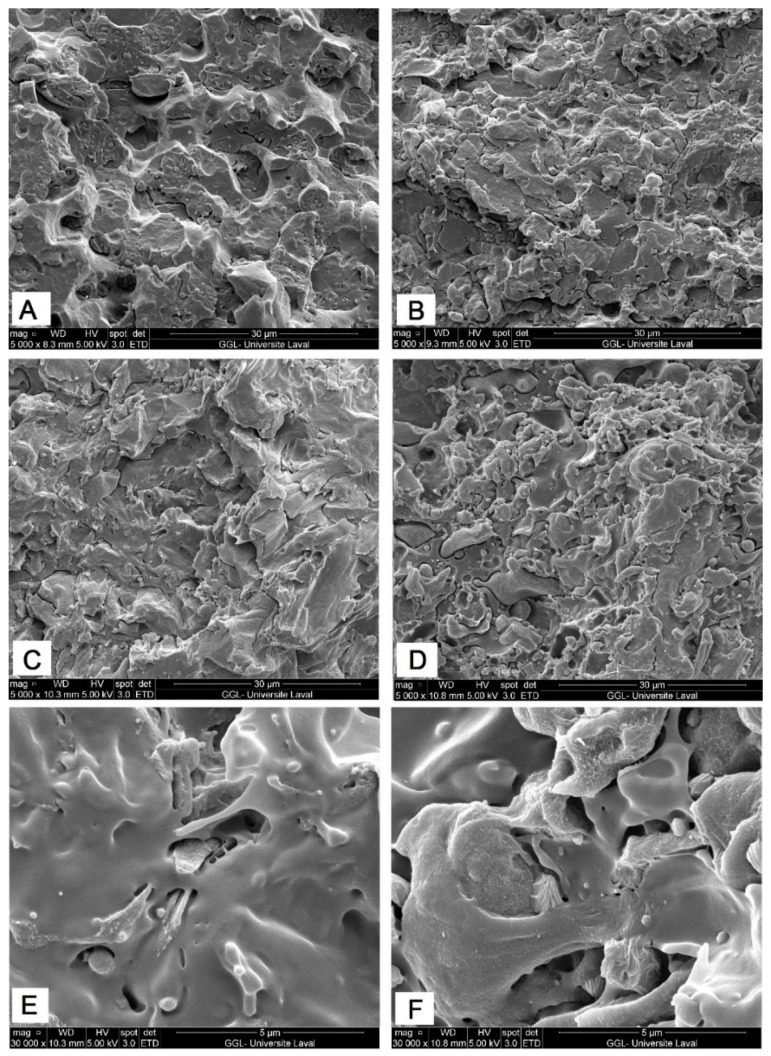
SEM micrographs of PA45/(PBAT10*) (**A**,**C**,**E**) and PA45(PEO10*) (**B**,**D**,**F**) ternary blends (see Section 3.2 for definition).

The incorporation of premade block copolymer elastomers, such as SAN-GMA, EMA-GMA or PEBA, is commonly used to compatibilize PLA-based blends, to adjust interfacial adhesion and stabilize the morphology of dispersed droplets against coalescence during polymer blending as well as improve the macroscopic properties of the final blends, such as impact strength and elasticity [19,20,22]. Here, SEM images (Figure 3) show that the morphologies match the spreading coefficient predictions for PLA/PBAT/PA11 and PLA/PEO/PA11 ternary blends. Figure 3 clearly shows differences in the morphology of these multiphase blends depending on which elastomer (PEO or PBAT) was added (10 or 20 wt %). It is well known that the breakup and coalescence of dispersed droplets are common phenomena in polymer blends processing, which can be minimized by decreasing the diameter of the dispersed phase and/or lowering the interfacial tension upon the addition of a third component (compatibilizer) if such copolymers can be located at the interface and stabilize the morphology of multiphase systems. Figure 3 and Figure 4 show a more homogenous structure for ternary systems compared to binary ones (Figure 2). This observation supports the thermodynamic predictions, as the positive value of *λ_PA11/PEO/PLA_* (0.56 mN/m) suggested that PEO should wet the interfacial region between PLA and PA11 (complete wetting), resulting in improved interfacial adhesion and the elimination of voids at the PLA/PA11 interface via morphology stabilization, thus preventing PA11 droplet coalescence. The adhesion between PLA and PA11 was improved by creating a thick/soft interphase of PEO particles around PA11 droplets, promoting a more uniform dispersion of spherical particles of the minor phase (Figure 3C,D). In agreement with this observation, Heshmati et al. [13] observed higher PLA chain mobility and better interfacial interaction with PA11, thus decreasing the dispersed phase size and limiting coalescence upon the addition of PEO (5%) in a PLA/PA11 (70/30) blend. They claimed that a plasticization/miscibility effect of PEO with PLA occurred by increasing the diffusion of PLA chains toward the interface due to higher free volume improving the interfacial interactions in these polymer blends [13].

Figure 4B,D show that increasing the PEO content from 10 to 20% enhanced the interfacial interactions and wetting of PEO with PLA and PA11. Compared to binary PL50/PA50 (Figure 2C,D), it is more difficult to detect the homogeneously distributed PA11 phase, while agglomerated PA11 domains are collapsed, and much more homogeneous dispersion is observed, as the PA11 phase is strongly embedded into the PLA matrix (Figure 4D,F). On the other hand, Figure 3A,B and Figure 4A,C present a clean and featureless fractured surface with a slightly more pronounced pull-out of PBAT and PA11 particles as well as a large number of cavities due to the stress concentration around the agglomerated phase as a consequence of weak interfacial adhesion due to the poor wetting ability of PBAT, especially for the PL50/PA50 system (Figure 4A,C). In a similar work, Zolali and Favis [30] observed the negligible plastic deformation of the PLA phase upon PBAT addition due to its low interfacial interactions with PLA and PA11. They also observed that PBAT poorly wet the PLA/PA11 interface compared to EMA-GMA, which generated a finer distribution of dispersed droplets with smaller sizes due to reactions between the epoxy groups of EMA-GMA with either PLA or PA11 (reactive compatibilization). Similar findings support the higher coalescence rate of PLA/PE/PBAT blends than that of PLA/PE/PHBV by about 5% [33], as PBAT addition was not able to prevent droplet coalescence and surround the dispersed phase/located between the other two polymers due to its weak partial wetting behavior in line with the interfacial energy and spreading coefficient measurements in Table 1. It can be concluded that similar to the effect of solid particles in Pickering emulsions, PEO as soft copolymers can effectively promote the compatibility between PLA and PA11. As the interface becomes better bonded with less interfacial gaps, the blends can better transfer the applied stresses as described later in terms of mechanical properties [30].

### 2.3. Mechanical Properties

#### 2.3.1. Tensile Properties

**Figure 5:**
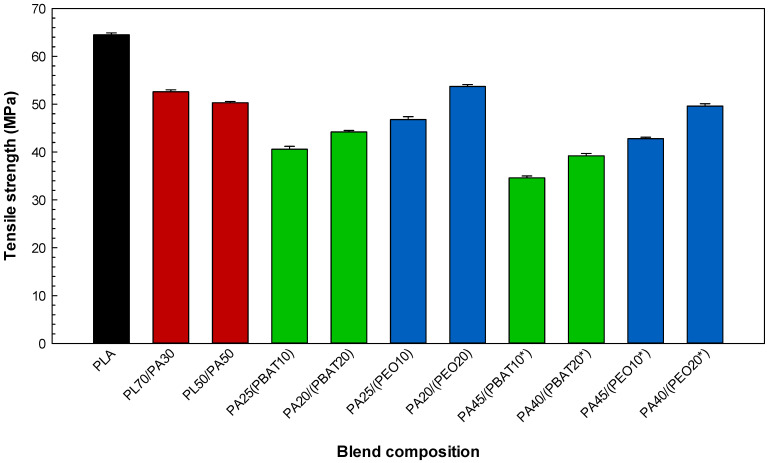
Tensile strength of the blends (see Section 3.2 for definition).

**Figure 6:**
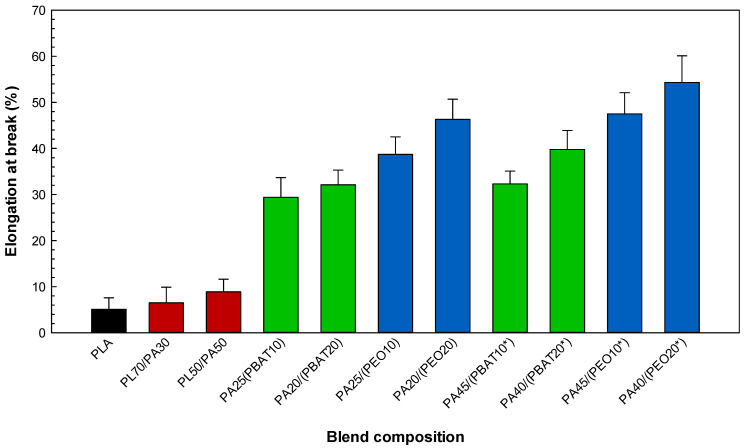
Elongation at break of the blends (see Section 3.2 for definition).

**Figure 7:**
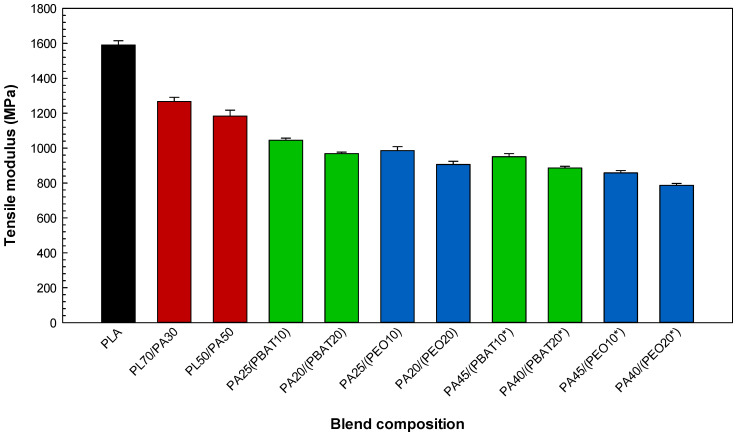
Tensile modulus of the blends (see Section 3.2 for definition).

The mechanical properties (tensile strength, elongation at break and tensile modulus) of the neat polymers (Table 2) as well as the binary PLA/PA11 and ternary PLA/PA11/elastomer systems (Figure 5, Figure 6 and Figure 7) are presented. As expected, Table 2 shows that PLA has a brittle behavior with high rigidity from its high modulus (1590 MPa) and low elongation at break (6.1%), while PA11 has lower rigidity (tensile modulus of 1024 MPa) and higher elongation (194.7%). The low tensile strength of the binary PLA/PA11 systems for both mixing ratios of 70/30 (56.4 MPa) and 50/50 (49.7 MPa) implies incompatibility and the presence of a poor and rigid interface failing to effectively transfer the stresses between the components, which is the weakest point leading to fracture [31]. The very low elongation (6.8%) of PL70/PA30 indicates that the brittle behavior of PLA plays a dominant role in the tensile properties of the blends (PLA is the predominant phase = matrix), while increasing the PA11 content up to 50% slightly increased the elasticity of PL50/PA50 to 7.9%.

According to the literature and in agreement with our tensile results for the ternary systems, it is often observed that the localization of an elastomer copolymer at the interface can convert glassy polymer blends into a much more deformable/ductile material having a distinct phase structure and mechanical properties [22,34]. Based on the results of the interfacial analysis (Table 1) and phase morphology (Figure 2, Figure 3 and Figure 4), ternary blends based on PEO have a finer morphology, leading to superior tensile properties compared to the blends containing PBAT (Figure 5). For the same blend composition, the higher tensile strength of the PA25/(PEO10) blend (49.6 MPa) compared to that of PA25/(PBAT10) with 46.8 MPa may be related to the possibility of dipole–dipole interactions between the ester groups of PLA and the ether groups of PEO as well as hydrogen bonding between the amide groups of PA11 and the ether oxygens of PEO, inducing better compatibility between PLA and PA11 and leading to the higher tensile strength of the corresponding sample [35,36]. According to the spreading coefficient predictions and SEM images, PEO is able to spread at the interface in PL50/PA50, providing a soft and deformable interfacial area or surrounding PA11 droplets in PL70/PA30, forming a core–shell structure (PA11 encapsulated by PEO) in the PLA matrix. This action promotes uniform particles distribution and consequently a better mixing state, leading to improved stress transfer between PLA and PA11 under tension (delayed fracture process). The ternary blends containing PEO yield higher deformation than PBAT due to the better interactions between PLA and PA11 and increased PLA chain mobility, as PEO addition resulted in a significant increase (104%) in elongation at break of PA40/(PEO20*) compared to that of PA40/(PBAT20*) with an elongation at break of 42.8% and PL50/PA50 with 7.9% (Figure 6). It has been reported that increasing the PLA chain mobility through the plasticization effect of PEO may lead to better interfacial adhesion and a more homogenous phase structure generating smooth stress transfer between PLA and PA11 as well as a homogenous structure with less particles coalescence and smaller size in PLA/PA11 blends. Heshmati and Favis [7] also observed substantial elongation at break increases for a PL50/PA50 blend from 6% to 100% after the addition of 10% PEO copolymer, which was attributed to its plasticization effect and the presence of a more elastic content (elongation at break of PEO = 516.4%, see Table 2) inducing higher deformation/elasticity, which was previously reported in similar works [37]. The decreasing trend of tensile moduli can be ascribed to the weaker intermolecular interactions of PLA upon PEO addition and improved rearrangement between polymer chains under external force to induce flexibility to PLA [38]. Figure 7 shows a decrease in the tensile modulus of the ternary blends upon addition of the elastomer phase with low inherent modulus (PEO = 73.6 MPa and PBAT = 105 MPa, see Table 2) and their very low glass transition temperatures (PEO = −67 °C [39] and PBAT = −30 °C [30]), which is typically reported for rubber-toughened plastic blends. For example, increasing the PEO content from 10 to 20 wt % showed a decreasing trend of tensile modulus for PL70/PA30 from 1167 to 1017 MPa and 946 MPa for the ternary systems attributed to the substitution of the rigid components (PLA and PA11) with a soft rubbery component (PEO) of low rigidity. In similar reports, Nofar et al. [40] observed important drops in the tensile modulus of PLA from 1800 to 1200–1250 MPa upon the addition of 25% PBAT or poly(butylene succinate-co-butylene adipate) (PBSA) (minor dispersed phases) due to the very low modulus of these elastomers, which is in line with the findings of Wu and Zhang [41] reporting significant decreases in PLA tensile modulus from 1.8 to 1.1 GPa upon the addition of 10% acrylonitrile–butadiene–styrene (ABS).

#### 2.3.2. Impact Properties

**Figure 8:**
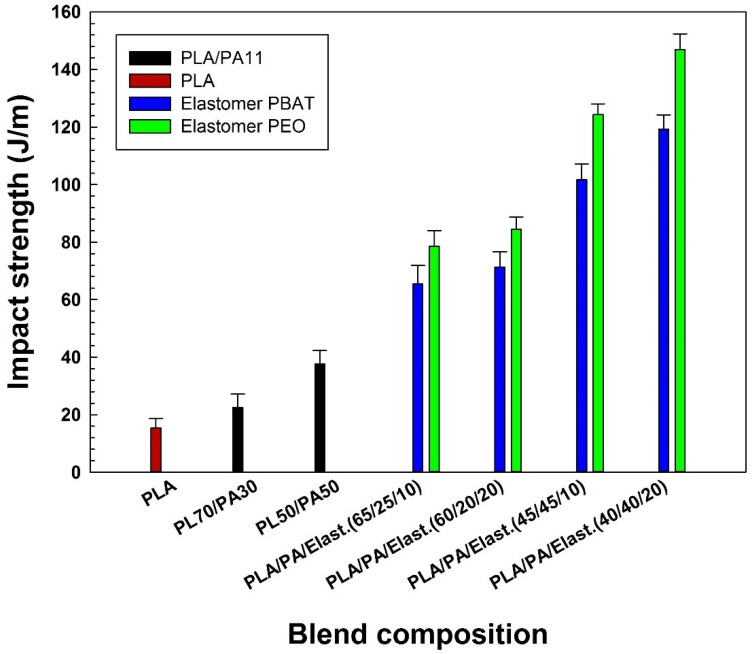
Impact strength of the blends (see Section 3.2 for definition).

The impact strength as a function of blend composition is presented in Figure 8. The concentration of elastomer (PEO and PBAT) was changed from 0 to 20 wt % in the ternary blends to examine its effect on interfacial interaction and impact strength. As expected, PLA, as a very brittle polymer, shows low impact strength (15.4 J/m). A partial miscibility of PLA with polyamide is reported in the literature due to hydrogen bonding between the ester groups of PLA and amine groups of polyamides. However, such interactions are not able to induce enough compatibility and good deformation to improve the impact strength of neat PLA/PA11 blends [15]. The blending of PLA and PA11, as very rigid polymers, results in the formation of a rigid interface that cannot smoothly transfer interfacial stresses in PLA/PA11 blends, resulting in easy crack initiation and propagation along these interfaces and leading to the low impact strength of the 70/30 (22.5 J/m) and 50/50 (37.4 J/m) blends. As expected, the notched Charpy impact strength follows the same trend as the elongation at break upon elastomer (PEO or PBAT) addition. The presence of an elastomer phase changes the rigid interface into a much more deformable area if the copolymer is localized at the PLA/PA11 interface, thereby increasing failure resistance through effective load transfer, especially when PEO is used [21]. The notched Charpy impact strength of PL70/PA30 and PL50/PA50 blends increases by 2.7 fold (from 22.5 to 84.5 J/m) and about 3 fold (from 37.4 to 147 J/m), respectively, with the addition of PEO (20 wt %). When PBAT is added to the PLA/PA11 blend, the ternary systems show slightly lower impact strength improvement with values of 71.4 J/m and 119.3 J/m upon the addition of PBAT (20 wt %) into PLA/PA11 systems at mixing ratios of 70/30 and 50/50, respectively. The theoretical values of interfacial tension measurements (Table 1) and morphological observations (Figure 2, Figure 3 and Figure 4) support phase debonding and the low increase in impact strength of the ternary blends modified with PBAT. In similar works, Zolali and Favis [30] reported that the impact strength of PLA/PA11 (50/50) increased from 15 to about 50 J/m upon the addition of 10% PBAT, triggering plastic deformation of the PLA and PA11 matrices, while Kanzawa and Tokumitsu [42] claimed that adding 18% PBAT as an impact modifier only increased the impact strength of PLA/polycarbonate (PC) (60/40) from 1.9 to 2.1 kJ/m^2^. Our results underline the relations between the phase structure and impact properties for the blends, which are directly influenced by the introduction of an elastomer. Here, the PA40/(PEO20*) system shows an impact strength (147 J/m) over 10 times higher than that of the virgin PLA (15.4 J/m), thus being considered as a super-tough material [43]. This behavior is in line with similar findings reporting a high level of interfacial interactions for PEO with PLA and PA11 leading to a homogenous phase morphology with fibrillated structures and percolation of the stress field around the cavitated PEO contributing to interfacial shear yielding of the matrix and the increased Izod impact strength of PLA/PA11 (50/50) from 17.3 to 58.3 J/m upon the addition of 20 wt % PEO [22].The presence of PEO as a plasticizer promotes PLA chain mobility and plastic deformation of the PLA matrix, thus increasing the Izod impact strength and elongation at break of binary blends by about 263% (from 11 ± 2 to 40 ± 8 J/m) and 15 fold (from 6 ± 1 to 100 ± 20 %), respectively [7].

More important increases in the impact strength of PLA/PA11/PEO systems can be related to the presence of partially wet PEO droplets spreading at the interface between PLA and PA11 phases modifying the stress state at the interface of the other two phases to locally release the triaxial stresses and contributing to plastic deformation [22]. It is of high importance to create internal rubber cavitation ahead of a crack and involve the matrix in the plastic deformation to delocalize the triaxial stress, leading to more impact energy dissipation, and to obtain rubber toughened plastic blends [18]. The PEO assembled at the interface between PLA and PA11 contributes to internal rubber cavitation; hence, a significant increase in the impact strength of the ternary blends is observed, while less cavitation is expected to occur upon PBAT addition [22,30]. A suitable interfacial adhesion, better wetting of the particles, finer droplets morphology and lower resistance to the cavitation (low moduli of 50 MPa) of PEO localized at the PLA/PA11 interface provides interfacial interactions to improve the shear yielding and postpone phase debonding or interfacial void formation, leading to significant impact strength improvement.

## 3. Materials and Methods

### 3.1. Materials

Table 3 summarizes the grades, melting point (*T_m_*), density at 25 °C, melt flow rate (MFI) at 210 °C, and chemical structure of all the materials used in this study. In all cases, the resins were used as received. Residual moisture was removed from the samples prior to the experiments using oven drying at 70 °C for at least 8 h except for PEO, which was dried at 40 °C.

### 3.2. Processing

Binary PLA/PA11 blends and ternary blends of PLA/PA11/elastomer (PEO or PBAT) were prepared via melt mixing in a co-rotating twin-screw extruder Leistritz ZSE-27 with an L/D ratio of 40 and 10 heating zones coupled to a circular die (2.7 mm in diameter). All the samples were prepared with a screw speed of 100 rpm to give a total flow rate of 3 kg/h under a temperature profile of 175/175/180/180/190/190/200/200/200/200 °C from the feed hopper to the die. The extrudates were quenched in a cold-water bath and pelletized using a model 304 pelletizer (Conair, Stanford, CT, USA) and dried (60 °C for 12 h) to eliminate any residual water before molding. Injection molding was performed using a PN60 (Nissei, Japan) injection molding machine with a temperature profile of 200–200–190–180 °C (nozzle, front, middle and rear). The mold had four cavities to directly produce standard geometries (Type IV of ASTM D638) and impact test bars (dimensions 12.7 × 63.5 × 3.2 mm^3^) for mechanical characterization. The injection pressure was adjusted (45 to 65 MPa) depending on the compound viscosity, while the mold temperature was fixed at 30 °C. Table 4 summarizes the samples prepared in this study and the ratio of each component for binary and ternary blends.

### 3.3. Characterization

#### 3.3.1. Contact Angle Measurements

An optical contact angle analyzer (OCA 15 Plus, Future Digital Scientific Corp., Westbury NC, USA) was used at room temperature to measure the contact angle of the materials based on the sessile drop method. Water and ethylene glycol were used as liquids to obtain the average values of five replicates for each sample. The surface tension values were calculated using the following equations [26]:

(3) $$\left(1+cos\theta_{x}\right)\gamma_{x}=4\left(\frac{\gamma_{x}{}^{d}\gamma^{d}}{\gamma_{x}{}^{d}+\gamma^{d}}+\frac{\gamma_{x}{}^{p}\gamma^{p}}{\gamma_{x}{}^{p}+\gamma^{p}}\right)$$

(4) $$\left(1+cos\theta_{y}\right)\gamma_{y}=4\left(\frac{\gamma_{y}{}^{d}\gamma^{d}}{\gamma_{y}{}^{d}+\gamma^{d}}+\frac{\gamma_{y}{}^{p}\gamma^{p}}{\gamma_{y}{}^{p}+\gamma^{p}}\right)$$

where *γ^d^* and *γ^p^* are the dispersion and polar components of surface tension, respectively (*γ* *=* *γ^d^* *+* *γ^p^*). In addition, $\theta_{x}$ and $\theta_{y}$ are the contact angles of the polymer with water and ethylene glycol, respectively.

#### 3.3.2. Morphological Observation

An Inspect F50 scanning electron microscope (SEM) (FEI, Hillsboro, OR, USA) was used at 15 kV to take micrographs and observe the interfacial adhesion quality between all the phases. The cryogenically fractured specimens in liquid nitrogen were coated with gold/palladium to be observed at different magnifications.

#### 3.3.3. Mechanical Testing

An Instron (Norwood, MA, USA) universal mechanical tester model 5565 was used to perform tensile tests according to ASTM D638 using a 5 kN load cell at a rate of 10 mm/min and room temperature. The average values of the tensile strength (*σ_Y_*), Young’s modulus (*E*) and elongation at break (*ε_b_*) were reported for five dog bone specimens (type IV) with 3 mm thickness for each formulation.

The notched Charpy impact strength was measured on a Tinius Olsen (Horsham, PA, USA) model 104 at room temperature according to ASTM D256 using 10 samples (60 × 12.7 mm^2^) for each composition. Before testing, all the samples were automatically V-notched on a Dynisco (Franklin, MA, USA) model ASN 120 m sample notcher 24 h before testing.

## 4. Conclusions

This work underlined the importance of the presence of an intermediate elastomeric phase to modify the interfacial area and interactions in multiphase systems and improve their properties. In this particular study, the mechanical properties (elasticity and toughness) of brittle PLA-PA11 systems were investigated by the addition of PBAT and PEO.

The results clearly indicated the importance of interfacial interaction and morphology development to produce super-tough PLA-based materials. The morphological results for the ternary PLA/PA11/elastomer blend strongly correlate with the theoretical prediction of interfacial values and spreading coefficients. A positive spreading coefficient of *λ_PA11/PEO/PLA_* = 0.56 mN/m and a lower interfacial tension of polymer pairs in PLA/PA11/PEO compared to PLA/PA11/PBAT suggested a complete wetting of the interface by PEO compared to the partial wetting of PBAT droplets. PEO tended to completely wet the interface of PLA and PA11, thus generating a soft and deformable interfacial area. It was found that PEO addition generated a more homogenous structure with few voids/defects at the PLA/PA11 interface, while the addition of PBAT led to poorly distributed droplets which did not prevent pull-out of the dispersed particles and the presence of a large number of interfacial voids due to the weak interfacial adhesion and poor wetting ability of PBAT.

A brittle-to-ductile transition was achieved for fully biobased PLA/PA11 blend upon the addition of PEO (20 wt %) between PLA and PA11 resulting in a smooth stress transfer at the interface, thus improving shear yielding and more deformation/elasticity as well as leading to improved energy absorption/dissipation before complete parts failure. The addition of PEO (up to 20 wt %) to the PLA/PA11 (70/30) and (50/50) blends increased the elongation at break of up to 45.6% and 104%, while the impact strength was improved by 2.7 fold (from 22.5 to 84.5 J/m) and 3 fold (from 37.4 to 147 J/m), respectively.

Finally, PEO was more effective than PBAT in modifying the interfacial interactions, phase morphology and mechanical properties of the immiscible PLA/PA11 blend because of its partial miscibility with PLA and better affinity toward PA11 as well as increased PLA chain mobility, which further enhanced the interfacial interactions with PA11.

## Figures and Tables

**Table 1:** Surface tension and interfacial tension values at 25 °C calculated from contact angle and spreading coefficients (all values are in mN/m).

Polymer	*γ*	*γ^d^*	*γ^p^*	Interfacial Tension	Spreading Coefficient (*λ*)	
PLA	39.7 ± 0.3	26.3 ± 0.2	13.4 ± 0.1	*γ*_PLA/PA11_ = 3.65 ± 0.7	*λ_ijk_ =* −2.71	*λ_jih_ =* −3.16
PA11	42.0 ± 0.4	35.4 ± 0.2	6.6 ± 0.2	*γ*_PLA/PBAT_ = 2.87 ± 0.6	*λ_jik_ =* −4.59	*λ_ijh_ =* −4.14
PEO	33.7 ± 0.2	25.1 ± 0.1	8.6 ± 0.1	*γ*_PA11/PBAT_ = 2.38 ± 0.7	*λ_ikj_ =* 0.56	*λ_ihj_ =* −1.6
PBAT	53.1 ± 0.3	40.1 ± 0.2	13.0 ± 0.1	*γ*_PLA/PEO_ = 1.17 ± 0.5	**Complete** **wetting**	**Partial** **wetting**
				*γ*_PA11/PEO_ = 2.10 ± 0.6	*PLA (i), PA11 (j), PEO (k) and PBAT (h)*	

**Table 2:** Tensile properties of the neat polymers.

Sample Code	Tensile Strength (MPa)	Tensile Modulus (MPa)	Elongation at Break (%)
**PLA**	64.5 (0.4)	1190 (25)	6.1 (2.5)
**PA11**	48.3 (0.3)	1024 (11)	194.7 (7.3)
**PBAT**	19.7 (0.5)	105 (7)	486.1 (11.5)
**PEO**	24.6 (0.3)	74 (5)	516.4 (9.1)

**Table 3:** Main characteristics of the materials used.

Polymer	Supplier	Grade	T_m_ (°C)	Density (g/cm^3^)	MFI (g/10 min)	Chemical Structure
**PLA**	Nature Works	2003D	175	1.24	6	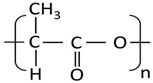
**PA11**	Arkema	Rilsan BMNO	178	1.03	11	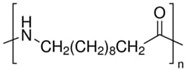
**PBAT**	TUHNE	TH801T	116	1.21	4.5	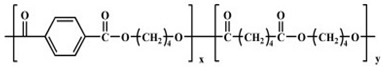
**PEO**	Dow Chemicals	Polyox WSR-N10	65	1.13	2.5	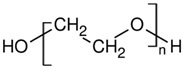

**Table 4:** Coding and formulation of the samples produced.

Sample Code	PLA (wt %)	PA11 (wt %)	PBAT (wt %)	PEO (wt %)
PLA	100	-	-	-
PL70/PA30	70	30	-	-
PL50/PA50	50	50	-	-
PA25(PBAT10)	65	25	10	-
PA20/(PBAT20)	60	20	20	-
PA25/(PEO10)	65	25	-	10
PA20/(PEO20)	60	20	-	20
PA45/(PBAT10*)	45	45	10	-
PA40/(PBAT20*)	40	40	20	-
PA45/(PEO10*)	45	45	-	10
PA40/(PEO20*)	40	40	-	20

## Data Availability

Not applicable.

## Sample Availability

Not available.
